# Effects of Acute Resistance Exercise with and without Whole-Body Electromyostimulation and Endurance Exercise on the Postprandial Glucose Regulation in Patients with Type 2 Diabetes Mellitus: A Randomized Crossover Study

**DOI:** 10.3390/nu13124322

**Published:** 2021-11-29

**Authors:** Roman Holzer, Benedikt Schulte-Körne, Jan Seidler, Hans-Georg Predel, Christian Brinkmann

**Affiliations:** 1 Department of Preventive and Rehabilitative Sport Medicine, Institute of Cardiovascular Research and Sport Medicine, German Sport University Cologne, 50933 Cologne, Germany; roman.holzer@protonmail.com (R.H.); b.schulte@dshs-koeln.de (B.S.-K.); jan.seidler96@web.de (J.S.); predel@dshs-koeln.de (H.-G.P.); 2 Department of Fitness & Health, IST University of Applied Sciences, 40233 Düsseldorf, Germany

**Keywords:** exercise, postprandial glucose regulation, continuous glucose monitoring, CGM, type 2 diabetes mellitus, T2DM, area under the curve (AUC)

## Abstract

Background: Long hyperglycemic episodes trigger complications in type 2 diabetes mellitus (T2DM) patients. Postprandial glucose excursions can be reduced by acute physical activity. However, it is not yet clear which type of exercise has the best effect on postprandial glucose levels. Methods: Six T2DM patients participated in three 20-min moderate-intensity exercise sessions after breakfast in a randomized order: resistance exercise with whole-body electromyostimulation (WB-EMS), resistance exercise without electromyostimulation (RES) and cycling endurance exercise (END). A continuous glucose monitoring system recorded glucose dynamics. Results: Postprandially-increased glucose levels decreased in all cases. Time to baseline (initial value prior to meal intake) was quite similar for WB-EMS, RES and END. Neither glucose area under the curve (AUC), nor time in range from the start of the experiment to its end (8 h later) differed significantly. A Friedman analysis of variance, however, revealed an overall significant difference for AUC in the post-exercise recovery phase (END seems to have superior effects, but post-hoc tests failed statistical significance). Conclusions: There are no notable differences between the effects of the different types of exercise on glucose levels, especially when comparing values over a longer period of time.

## 1. Introduction

Diabetes mellitus is a disease that affects over 450 million people worldwide with 90–95% affected by type 2 diabetes mellitus (T2DM) [1]. Obesity and physical inactivity play a critical role in its development [2]. Regular aerobic exercise of at least 150 min per week at moderate to high intensity is recommended in the treatment of T2DM [3,4]. However, physical activity should preferably not be limited to endurance-based training but should be combined with resistance training [4]. Resistance exercises for major muscle groups should be performed two to three times a week [3]. Muscle contractions during exercise lead to a translocation of glucose transporters within the muscle cell via an insulin-independent mechanism [5]. This process corresponds to the physiological effect of insulin and induces a reduction in blood glucose levels [5]. Both insulin sensitivity and glucose tolerance can be increased up to 24 h or more following physical exercise [6].

While implementing these recommendations in daily life, people with T2DM face several barriers, however, including lack of time [7,8]. Originally established in competitive sports, electromyostimulation (EMS) training has become increasingly popular in health and recreational sports. One major advantage of EMS training is the time saved compared to conventional resistance training [9,10]. EMS training has proved to be successful for improving physical performance [9,10,11,12]. Through EMS, muscles are stimulated percutaneously by electrical stimulation via electrodes on the skin surface. A distinction in the use of EMS depends on whether its application is combined with voluntary contractions/active exercises or is applied passively during physical rest. In general, the combination of EMS with voluntary contractions/active exercises induces greater muscular adaptations than the passive application of EMS or solely voluntary contractions without EMS [12]. Another distinction in EMS training is made between local stimulation, where the focus is on a single muscle only, and whole-body EMS (WB-EMS), where entire muscle groups are used for a more functional training. The frequency of the applied stimulation current influences different muscle fibers. That is, high-frequency stimulation currents are used to increase maximum strength, while low-frequency currents can lead to an improvement in strength endurance [9].

Increased muscle contraction due to percutaneous electromyostimulation can result in higher muscle fiber damage if used excessively [13,14]. Increased muscle fiber damage, which can occur with conventional exercise as well, can result in elevated creatine kinase (CK) levels [14,15]. Consequently, WB-EMS should be used at moderate intensity when applied for the first time to prevent adverse health effects [16]. However, when it is adequately and properly instructed, WB-EMS is considered a safe and efficient training method, even for physically untrained individuals [10].

As resistance exercise with WB-EMS is offered by several providers in both commercial and clinical settings, it is important to determine its effects on patients’ glucose regulation in relation to the effects of well-established and recommended conventional forms of exercise (resistance and endurance type). Therefore, the main objective of the present study is as follows:

Evaluation of the effects of resistance exercise with WB-EMS compared to the effects of conventional resistance exercise (without WB-EMS) and endurance exercise on the postprandial glucose regulation in patients with T2DM.

The glucose profiles of the different exercise modes should be recorded using a continuous glucose monitoring (CGM) system.

As a secondary objective of the study, the subjects’ perception of the EMS application, as well as of their use of the CGM system and awareness of their individual glucose responses, will be analyzed using a questionnaire.

## 2. Materials and Methods

This randomized crossover study with three exercise sessions was conducted in accordance with the Good Clinical Practice guidelines and the Declaration of Helsinki. All subjects performed three moderate-intensity exercise bouts (resistance exercises with whole-body electromyostimulation: WB-EMS; resistance exercises without whole-body electromyostimulation: RES; continuous endurance exercise: END) in a randomly assigned order. The project was approved by the Ethics Committee of the German Sport University Cologne (reference number 003/2020).

### 2.1. Participants

Non-insulin-dependent T2DM patients without severe nephropathy, neuropathy, advanced retinopathy and/or severe cardiovascular complications were included in this study. Recruitment took place via leaflets in diabetes-related surgeries, via social media (diabetes professional associations, diabetes support groups) and through newspaper articles.

The decision on inclusion/exclusion was based on a detailed anamnesis, completion of a medical examination and recommended contraindications for non-medical WB-EMS use [17]. As this study was conducted under medical supervision and with medically trained investigators, diabetes mellitus was not considered as an absolute contraindication. The participants’ individual pharmacological therapies were not modified during the study. All subjects participated voluntarily and provided written informed consent for inclusion in the study.

### 2.2. Study Design

The subjects visited the institute on 4 days with a break of at least 72 h. The visits entailed a preliminary visit for anamnesis, preparation and familiarization, and three exercise sessions. On the day of anamnesis, the subjects underwent a medical examination, followed by an incremental exercise test (IET) to determine their individual maximum performance level. The IET was carried out on a cycling ergometer according to the World Health Organization (WHO) protocol (25 watts + 25 watts every 2 min) until exhaustion or the occurrence of predefined stopping criteria [18]. For the glycemic assessment, a continuous glucose monitoring (CGM) system was attached to the subjects’ left upper arm. To give the participants sufficient time to completely recover, and as the CGM’s accuracy might be reduced in the initial days of use [19,20], a run-in phase of at least 72 h was scheduled between the first and second visit. At the end of the first visit, a familiarization session with the WB-EMS was performed, determining the size of the vest and pants and the intensity of the EMS stimulus. In addition, the subjects were shown how to perform the exercises.

**Figure 1:**
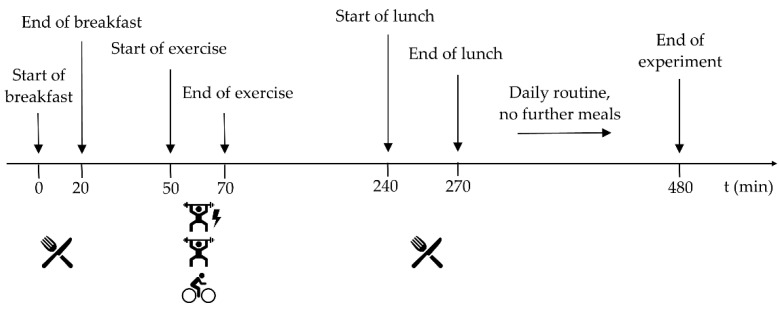
Standardized schedule of the experiment.

On the following days of the experiment, the exercise sessions were conducted in a randomized order according to a standardized daily schedule (Figure 1). A venous blood sample was collected to determine the subjects’ blood variables, namely fasting glucose, glycated hemoglobin (HbA1c), total cholesterol, high-density lipoprotein (HDL), low-density lipoprotein (LDL) and triglycerides.

### 2.3. Experimental Procedure

The participants arrived at the institute in the early morning after an overnight fast. The subjects were given a standardized breakfast (310 kcal, 61.5 g carbohydrates (of which 61.5 g was sugar), 0.5 g fat, 14.5 g protein), which they were asked to consume within 20 min. The meal contained a high amount of sugar to induce a significant glucose excursion in the subjects.

Fifty min after the start of the measurements, subjects started exercising for 20 min (assigned to the different exercise types in a randomized order). After the exercise, the participants remained in a state of physical rest. They had lunch exactly 4 h after the start of the experiment. It included a standardized meal (625 kcal, 77.5 g carbohydrates (of which 7.0 g was sugar), 23.5 g fat, 22.5 g protein), which they were asked to consume within 30 min.

Subsequently, the subjects were allowed to leave the institution, but were instructed to stick as close to their daily routine as possible and not engage in any further physical activities. Furthermore, no further food intake was allowed for the subsequent 4 h.

### 2.4. Exercise Sessions

Since resistance training with EMS can lead to strong muscular strain, the recommended EMS exercise duration was limited to a maximum of 20 min [16]. To compare glucose responses of the different exercise types, the three exercise sessions were matched for exercise time.

The protocol for the resistance exercise (RES) session was identical to the WB-EMS session, with the exception that no WB-EMS was applied. RES was used to evaluate the direct effect of EMS.

In total, the subjects performed 8 exercises (squats, biceps curls, good mornings, bilateral shoulder horizontal abductions, lunges left, lunges right, bench presses, crunches) with one set of 20 repetitions each (except crunches with 10 repetitions) over the 20 min. The exercises were performed one after the other without a break between sets.

Some exercises (squats, good mornings, lunges, crunches) were performed as body weight exercises (the line of pull was perpendicular to the ground). An elastic resistance band was used for all other exercises (biceps curls, bilateral shoulder horizontal abductions and bench presses).

The intensity of the exercises could be adjusted by changing the range of motion or by using different resistance bands. Participants were instructed to perform the exercises with moderate intensity, so that they would have been able to perform 3–4 more repetitions after completion of each set (until, in theory, muscular failure would have been occurred).

The EMS device XBody Newave (XBODY International Kft., Budapest, Hungary) was used for the WB-EMS session. The bipolar, low-frequency stimuli (80 Hz) were applied percutaneously via vest and pants to the respective muscle groups.

Electrical stimulation was applied for 4 s each time, within which one repetition was performed, followed by 4 s of rest. The intensity of the electrical stimulation was determined by the participants and compared with a muscle contraction scale (0–10; 0 = no contraction, 10 = extremely strong muscle contraction), while participants were asked to stay at a level of 4–5 (mild to moderate muscle contraction). Similar protocols have been used in former research studies [9,10,21] and are also being used in commercial EMS studios.

Moderate-intensity continuous endurance exercise (END) was performed on a bicycle ergometer at 50% of the maximum power achieved at IET. This training modality for END is commonly used in training therapy and research [22,23].

### 2.5. Continuous Glucose Monitoring

Participants’ glucose responses were measured using the FreeStyle Libre 2 (FSL2) Flash Glucose Monitoring System (Abbott Diabetes Care, Witney, Oxon, UK). FSL2 is a measurement device that continuously monitors glucose in the interstitial fluid (ISF) of the subcutaneous adipose tissue. The system consists of a reader and a sensor that is worn on the upper arm. The sensor is attached with an applicator, whereby a flexible, sterile tip is inserted in the subcutaneous tissue and fixed with an adhesive foil. In the EU, the FSL2 has been approved for a 14-day use. During this period, the measured value can be scanned as often as desired. A value is also automatically saved in the system for every 15 min up to 8 h. The sensor does not require frequent calibration as the calibration process is part of the manufacturing process [24]. The system is ready for use 1 h after the sensor’s application. The accuracy of readings on the first day(s) is lower than on subsequent days, however, due to temporary local trauma at the sensor’s application site [19].

### 2.6. Questionnaire

Subjects were asked to complete a questionnaire before and after participation in the study. The questionnaire was designed to collect additional information on the participants’ expectations and experiences. All “questions” were expressed as statements, and agreement/disagreement was assessed using a 5-point Likert scale (“agree”, “rather agree”, “neither nor”, “rather disagree”, “disagree”).

### 2.7. Data Analysis

The continuous glucose values of FSL2 were exported as a .csv-file and linearly interpolated to one value per minute.

The time interval from the start of the experiment until the postprandial glucose levels returned to baseline for the first time (time to baseline = TTB) was determined.

To allow for a comparison of the individual exercise sessions despite different starting values, we calculated the change from the starting value (Δglucose) for each glucose profile.

The area under the curve (AUC) was calculated for each minute value using the trapezoidal rule [25]. Thus, the AUC of the single minute values could be summed up for different time frames (Table 1).

An important parameter for assessing health risks in diabetes mellitus is the time in target range (TIR), expressed as the percentage of readings/time within the glucose range of 70–180 mg/dL [26,27]. Time above target range (TAR) as well as below target range (TBR) were also calculated.

The nonparametric Friedman test was used to compare differences between conditions. If overall significant differences were found, Dunn–Bonferroni post-hoc tests were performed to explore which data from which exercise types differed from each other.

Results from the questionnaire were analyzed using the Wilcoxon signed-rank test, where appropriate.

The results are presented as mean values (± standard deviation (SD)). Statistical significance was considered at *p* < 0.05. Data were analyzed using Microsoft Office Excel 2016 for Windows (Microsoft Corporation, Redmond, WA, USA) and IBM SPSS Statistics 27 (IBM Corp., Armonk, NY, USA). Illustrations were made with Microsoft Office Excel 2016.

## 3. Results

### 3.1. Subjects’ Characteristics

A total of 6 subjects (3 women, 3 men) with T2DM were enrolled in the trial. On average, T2DM was first diagnosed in the patients 5.3 (±3.3) years before the start of the investigation. Anthropometric data and metabolic variables of the participants are presented in Table 2.

### 3.2. Glucose Profiles

The mean fasting glucose was 137.5 (±14.3) mg/dL for WB-EMS, 137.4 (±17.0) mg/dL for RES and 149.8 (±34.1) mg/dL for END at the beginning of the experiment. Postprandial peak values after breakfast were reached, on average, at 56 min (WB-EMS), 51 min (RES) and 49 min (END), which was quite similar between the different conditions. Glucose levels decreased with the beginning of physical activity and during the following passive recovery period. The mean postprandial minimum was reached just before the start of lunch after 237 min (WB-EMS; 103.2 ± 14.5 mg/dL), 233 min (RES; 108.1 ± 11.2 mg/dL) and 243 min (END; 107.1 ± 13.1 mg/dL). Glucose levels over the subsequent 4 h during which the subjects followed their daily routines averaged at 147.2 (±26.1) mg/dL for WB-EMS, 151.1 (±14.8) mg/dL for RES and 148.2 (±26.3) mg/dL for END. The mean glucose levels for the entire 8-h experimental period were 146.9 (20.5) mg/dL for WB-EMS, 149.2 (15.6) mg/dL for RES and 146.4 (20.1) mg/dL for END.

All of the above-mentioned values did not significantly differ between the three experimental conditions (WB-EMS, RES, END).

### 3.3. Time to Baseline (TTB)

After glucose excursions were induced by breakfast at the beginning of the experiment, physical activity was performed to lower postprandial glucose levels. To assess the rate of glucose reduction, we calculated the time until subjects reached their baseline level (initial value before breakfast) for the first time. The TTB determined for WB-EMS was 120.7 (±36.9) minutes, for RES it was 127.3 (±40.3) minutes and for END it was 100.8 (±42.4) minutes. There were no significant differences between the three conditions.

### 3.4. AUC Analyses

**Figure 2:**
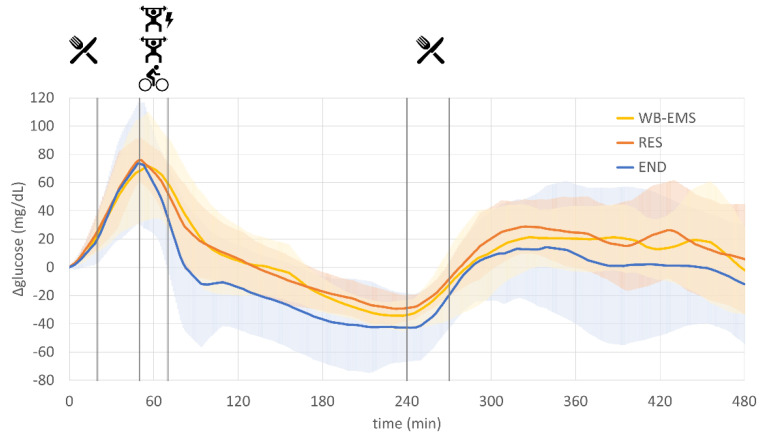
Rate of glucose changes (Δglucose) for resistance exercise combined with whole-body electromyostimulation (WB-EMS), resistance exercise without WB-EMS (RES) and endurance exercise (END).

As the fasting glucose levels at the beginning of each experimental run differed not only inter- but also intraindividually, the change from the initial baseline value (Δglucose) was calculated for better comparability. This resulted in normalized glucose curves starting from 0, which are illustrated in Figure 2.

**Figure 3:**
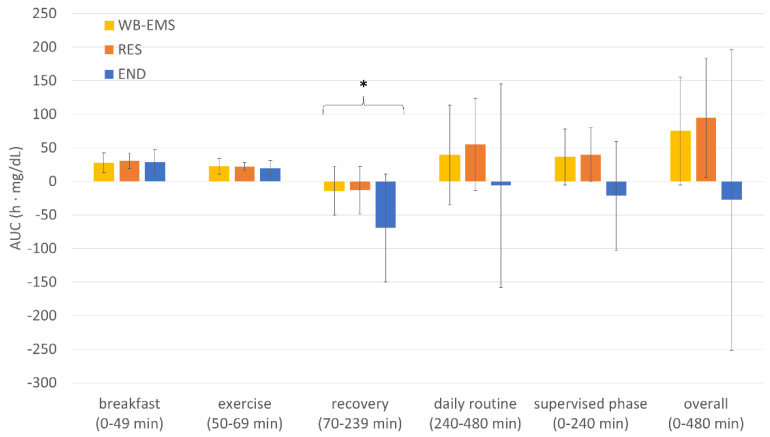
Area under the Δglucose curve (AUC) of the different experimental phases. Values are means ± SD. WB-EMS, resistance exercise combined with whole-body electromyostimulation; RES, resistance exercise without WB-EMS; END, endurance exercise. * Significant difference between conditions (overall significance: *p* = 0.042, post-hoc tests failed significance).

The area under the respective Δglucose curves was calculated for the AUC analyses. AUC values of different time intervals are presented in Figure 3.

During the recovery phase (70−240 min), a significant overall difference was found between the three exercise methods (*p* = 0.042). The Dunn–Bonferroni post-hoc tests did not, however, reveal a significant result (END vs. WB-EMS: *p* = 0.063; END vs. RES: *p* = 0.130. WB-EMS vs. RES: *p* = 1.000).

### 3.5. Time in/above/below Range (TIR, TAR, TBR)

Within the investigated 8-h periods, mean TIR was 80.8% (±15.6%) for WB-EMS, 83.8% (±14.5%) for RES and 79.0% (±19.3%) for END. While none of the subjects spent time below target range (TBR), the results for time above target range (TAR) were 19.2% (±15.6%) for WB-EMS, 16.2% (±14.4%) for RES and 21.0% (±19.3%) for END. There were no significant differences between the three conditions.

### 3.6. Questionnaire

**Figure 4:**
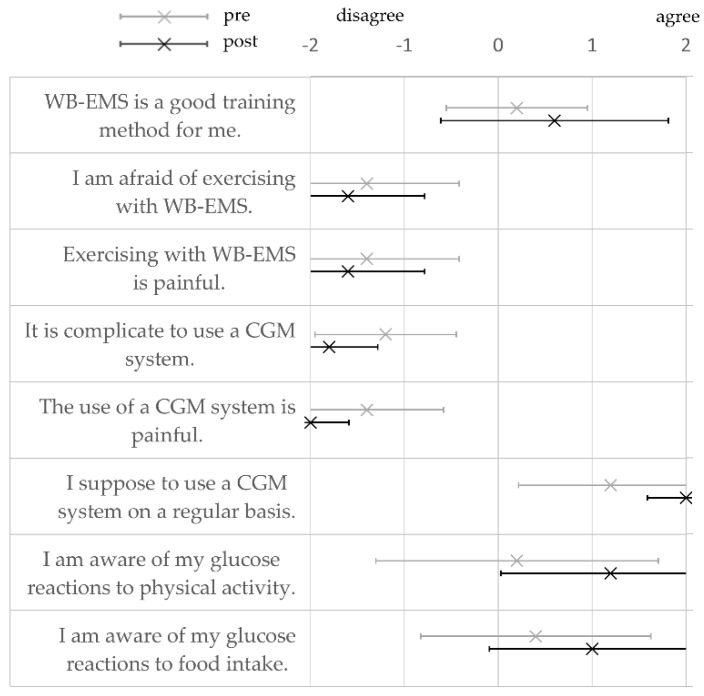
Subjects’ rating based on their perception of the whole-body electromyostimulation application (WB-EMS), the use of the continuous glucose monitoring (CGM) system and glucose awareness pre- and post-study participation. Values are means ± SD.

Subjects’ agreement with the statements in the questionnaire was coded as follows: −2 = disagree, −1 = rather disagree, 0 = neither nor, 1 = rather agree, and 2 = agree. The mean values are shown in Figure 4. Differences between pre- and post-survey were not significant.

WB-EMS was tendentially rated as a good training method and participants were not afraid of exercising with WB-EMS. After participation in the study, all subjects rated exercising with WB-EMS as (rather) pain-free. Furthermore, everyone agreed that the use of a CGM system is (rather) uncomplicated and well tolerable. Glucose awareness both during physical activity and after food intake tended to increase as a result of participation in the study. There were no significant differences in the ratings post- versus pre-study participation.

## 4. Discussion

In this study, exercise was shown to be effective in lowering postprandial glucose levels. This result is in line with observations from other studies [28,29]. The glucose-lowering effect was observed for all types of exercise investigated, even at an exercise duration of 20 min.

Theoretically, WB-EMS, with its high recruitment of glycolytic type 2 fibers [30] and increased muscle contraction [12], was expected to result in a greater decrease in postprandial glucose levels due to contraction-induced glucose uptake [5,31]. In this study, however, no significant difference in the acute postprandial glucose response was found between exercise with and without WB-EMS.

In former studies, increased postprandial glucose uptake was observed from passive EMS application at the quadriceps femoris (at rest without exercise/voluntary contraction) compared with the control condition without EMS [32,33].

Long-term studies showed improvements in insulin sensitivity, glycemic control or physical performance after several weeks of local or whole-body EMS [34,35,36,37,38] with both active and passive application.

To date, the acute effects of WB-EMS application on postprandial glucose regulation has not been investigated in comparison with different exercise types without EMS, as was performed in this study.

Some potential reasons might explain the absence of significant differences between the effects of WB-EMS and RES or END on postprandial glucose regulation in this study.

Although the subjects completed a familiarization session with the WB-EMS during their first visit, they were largely inexperienced in exercising with electrical muscle stimulation. Consequently, the WB-EMS exercise intensity was initially set at a moderately intense level, according to the general recommendations for WB-EMS use in untrained persons [16]. Higher intensities when exercising with WB-EMS should only be used after several training sessions, as very high intensities could lead to severe muscle fiber damage and rhabdomyolysis, especially in untrained persons [10,13]. However, enhanced carbohydrate oxidation has been reported for EMS applications also with low intensity [30].

Furthermore, electrical stimulation during exercise as used in WB-EMS, can lead to higher acute serum cortisol levels compared to exercise without electrical stimulation [39]. Cortisol as a glucocorticoid promotes gluconeogenesis in the liver, on the one hand, and reduces skeletal muscle glucose uptake, on the other [40]. It can be speculated that these two effects of cortisol counterbalanced the possible effects of increased contraction-induced glucose uptake and increased carbohydrate oxidation induced by WB-EMS. Unfortunately, cortisol levels were not measured in the present study, but should be considered in future studies for further clarification.

While subjects performed different exercises with defined repetitions and breaks between them in WB-EMS and in RES, they continuously performed moderately intense activity in END. It can be concluded with caution that the steady movement pattern of END is more suitable than resistance exercise for reducing postprandial glucose levels in the subsequent recovery period. These findings are consistent with results from other studies that investigated the effects of resistance versus endurance exercise on postprandial glucose response, where advantages were identified for endurance-based exercises [28,41,42].

During exercise, skeletal muscle glucose uptake and consumption depend predominantly on the intensity and duration of exercise [43,44]. The duration of the exercise sessions was identical, and the intensities were in a comparable range. However, glucose uptake is not only elevated during exercise but also in the post-exercise period due to glycogen repletion, increased GLUT4 translocation and enhanced insulin sensitivity [4,31,43,44]. Continuous motion during endurance exercise is likely to elicit higher glucose uptake in the subsequent recovery phase than resistance exercise [28,41,42]. One reason for this difference may be the increased muscle damage after resistance exercise, especially after high-intensity and/or eccentric exercises, resulting in decreased GLUT4 content and decreased insulin signaling in the damaged muscle [45]. Increased muscle damage may also occur during training with WB-EMS due to percutaneous stimulation [13,14]. In the long term, however, resistance training appears to have comparable positive effects on glycemic control in T2DM than endurance-based training [4,28,29,46].

Increased skeletal muscle glucose uptake and insulin sensitivity can be elevated up to 24 h or longer following exercise [4,6]. During the subjects’ daily routine after lunch, no AUC differences were observed between the three exercise types explored in the present study. Although a difference in the recovery phase was expected to have manifested in the following hours, this was not the case. It could be that the effects of the different exercise sessions equalize over time, which would also explain the comparable long-term effects of resistance and endurance training [4,28].

In addition to the experiments, a survey on the participants’ expectations and experiences was carried out. The survey indicated that the participants had no apprehension of exercising with WB-EMS as an innovative training method. Subjects obviously did not experience any pain during the WB-EMS training sessions. The high degree of supervision with a maximum of two subjects per trainer/coach could have had an appealing effect and might motivate people with T2DM and low affinity to conventional exercise to engage in this type of exercise [10,38].

The use of a CGM system was rated as very positive by the subjects. Studies have demonstrated positive effects on glycemic control by using CGM systems, also in T2DM patients [47].

### Strengths and Limitations

The study´s strengths include: randomized-controlled design, standardized process and data recording over the day as well as inclusion of a special patient group.

The study was performed in an exploratory manner. One limitation of this study is the small sample size, although the number of participants in this study is comparable with that of other studies in this field [30,32,33,48,49].

EMS training is costly in a commercial setting. For this reason, it is not possible for many patients to use this type of training. However, the EMS market is constantly growing and diverse sports and exercise offers increase the chance for an individual with T2DM to find an offer that is suitable for him/her. Therefore, training recommendations for T2DM patients should also consider this type of training. Ultimately, this study shows that moderate-intensity physical activity, regardless of resistance exercise (with or without EMS) or endurance exercise, can lower glucose levels in T2DM patients.

## 5. Conclusions

The results of this preliminary study suggest that all types of exercise (WB-EMS, RES, END) affect postprandial glucose concentrations similarly. There were no notable differences between their effects on glucose levels, especially when comparing values over a longer period of time. WB-EMS resistance exercise as an innovative type of exercise was well tolerated by the patients.

The lack of additional beneficial effects of electromyostimulation (when used in resistance exercises) on glucose regulation throughout the day may be due to the acute exercise setting and further studies on chronic use may be required to elucidate the potential beneficial effects of WB-EMS resistance training for T2DM patients.

Results of this study could also be helpful for patients treated with insulin to better assess if/to what extent insulin doses/extra carbohydrate doses need to be adjusted.

## Figures and Tables

**Table 1:** Experimental phases and corresponding time intervals.

Phase	Interval	Start	End
breakfast	0–49 min	beginning of experiment	start of exercise
exercise	50–69 min	start of exercise	end of exercise
recovery	70–239 min	end of exercise	start of lunch
daily routine	240–480 min	start of lunch	end of experiment
supervised phase	0–240 min	beginning of experiment	start of lunch
overall	0–480 min	beginning of experiment	end of experiment

**Table 2:** Subjects’ characteristics.

Anthropometric Data		Metabolic Variables	
age (years)	55.2 ± 7.5	HbA1c (%)	7.1 ± 0.5
height (cm)	175.4 ± 5.9	fasting blood glucose (mg/dL)	169.0 ± 49.3
weight (kg)	100.2 ± 21.6	total cholesterol (mg/dL)	202.5 ± 53.1
BMI (kg/m^2^)	32.7 ± 7.6	LDL (mg/dL)	117.8 ± 48.4
		HDL (mg/dL)	48.5 ± 10.8
		triglycerides (mg/dL)	208.7 ± 84.3

Values are means ± SD. BMI, body mass index; HbA1c, glycated hemoglobin; LDL, low-density lipoprotein; HDL, high-density lipoprotein.

## Data Availability

Data are available from the corresponding author upon reasonable request.
